# Supplementary material for: Managing Super Pests: Interplay between Pathogens and Symbionts Informs Biocontrol of Whiteflies
Source: Microorganisms. 2024 Apr 28;12(5):887. doi: 10.3390/microorganisms12050887 (PMC11123976; doi:10.3390/microorganisms12050887)
Supplement: Supplementary file 1 [file microorganisms-12-00887-s001.zip › Table S1.pdf]

TABLE S1 | Summary of high-throughput sequencing reads analysis, microbial community diversity richness (OTUs, 97%), sample coverage (Good's coverage), diversity index (Shannon, ACE, Shannon, Simpson), and estimated OTU richness (Sobs, Chao1) for community diversity analyses of 48 samples from whitefly of different development treatments.

Sample	Seq_num	Base_num	Mean_length	Alpha diversity estimators					
				Sobs	ACE	Chao	Shannon	Simpson	coverage
N_C1	39343	16311555	415	44	62.56	59.00	1.04	0.4261	0.9996
N_C2	39499	16355728	414	48	66.46	61.91	1.04	0.4363	0.9995
N_C3	36895	15239950	413	57	77.12	76.00	1.07	0.4532	0.9994
N_C4	43538	18172823	417	47	63.81	62.00	1.11	0.3820	0.9996
N_C5	46427	19177495	413	44	60.35	57.13	0.98	0.4707	0.9997
N_C6	48339	20047154	415	56	80.43	84.88	1.05	0.4205	0.9995
N_LC$_{20}$1	45106	18735860	415	30	50.47	39.17	1.17	0.3753	0.9997
N_LC$_{20}$2	47677	19766506	415	33	47.07	55.75	1.13	0.4002	0.9997
N_LC$_{20}$3	47289	19710672	417	29	51.13	38.17	1.12	0.3723	0.9998
N_LC$_{20}$4	52161	21742589	417	27	32.79	31.67	1.14	0.3624	0.9998
N_LC$_{20}$5	48466	20114002	415	31	45.36	44.20	1.07	0.4094	0.9997
N_LC$_{20}$6	43787	18225334	416	26	40.74	39.75	1.06	0.3997	0.9997

$N\_LC_{30}1$	46897	19413115	414	65	85.44	98.00	1.13	0.4187	0.9995
$N\_LC_{30}2$	48877	20188833	413	31	112.32	51.00	0.93	0.4769	0.9997
$N\_LC_{30}3$	48705	20088368	412	21	136.11	54.00	0.95	0.4891	0.9997
$N\_LC_{30}4$	45347	18734348	413	35	88.30	45.50	0.95	0.4713	0.9996
$N\_LC_{30}5$	72545	29957049	413	58	68.67	71.33	1.04	0.4558	0.9998
$N\_LC_{30}6$	47340	19581404	414	19	31.35	28.33	1.07	0.4307	0.9998
$N\_LC_{50}1$	46744	19399945	415	39	94.22	54.00	1.12	0.3935	0.9996
$N\_LC_{50}2$	44051	18117849	411	52	72.44	65.91	0.91	0.5294	0.9996
$N\_LC_{50}3$	48963	20200701	413	49	102.41	77.88	0.99	0.4801	0.9995
$N\_LC_{50}4$	49775	20640993	415	53	71.27	63.20	1.11	0.4062	0.9996
$N\_LC_{50}5$	50119	20773604	414	53	74.92	87.20	1.06	0.4240	0.9996
$N\_LC_{50}6$	51675	21408869	414	72	96.85	99.60	1.13	0.4048	0.9995
V_C1	41032	17169900	418	104	110.19	111.58	1.12	0.4032	0.9996
V_C2	39781	16736712	421	35	149.26	98.33	0.97	0.4382	0.9995
V_C3	45926	19070156	415	51	62.99	57.50	0.92	0.4534	0.9997
V_C4	40859	16997509	416	49	69.57	62.91	0.95	0.4394	0.9995
V_C5	45176	19032601	421	35	167.71	70.00	0.96	0.4346	0.9995

V_C6	42344	17686742	418	35	59.26	69.00	0.97	0.4191	0.9996
V_LC$_{20}$1	44682	18769921	420	54	56.05	55.43	1.01	0.4428	0.9999
V_LC$_{20}$2	45875	19090659	416	49	56.35	53.58	0.97	0.4351	0.9998
V_LC$_{20}$3	51131	21225810	415	51	63.48387	64.125	1.006784	0.429875	0.999701
V_LC$_{20}$4	52679	21716711	412	45	61.97465	57.36364	0.829962	0.54094	0.999654
V_LC$_{20}$5	49432	20695671	419	66	73.58166	72.5	1.085446	0.398266	0.999729
V_LC$_{20}$6	48872	20193803	413	44	56.8004	51.33333	0.937092	0.47917	0.999751
V_LC$_{30}$1	42010	17443350	415	49	59.03536	54.5	1.025444	0.419469	0.999703
V_LC$_{30}$2	42685	17659350	414	54	140.0637	77.21429	0.950345	0.46851	0.999341
V_LC$_{30}$3	42067	17599937	418	70	81.39167	76.5	1.405774	0.362719	0.999651
V_LC$_{30}$4	43763	18205706	416	45	61.42105	55.5	1.001857	0.4222	0.999646
V_LC$_{30}$5	42453	17592302	414	56	65.55952	69	1.040646	0.429633	0.999663
V_LC$_{50}$1	44034	18303121	416	63	78.53329	73.46154	1.050162	0.427452	0.999627
V_LC$_{50}$2	46525	19300100	415	85	96.4536	94.24077	1.273767	0.3944	0.999647
V_LC$_{50}$3	46192	19131357	414	63	79.53402	74.33333	0.616976	0.721626	0.999598
V_LC$_{50}$4	42864	17482391	408	73	89.07528	82.5625	1.033261	0.42964	0.999574
V_LC$_{50}$5	42822	17800621	416	84	94.54658	94.46154	1.187558	0.372654	0.999548

V_LC$_{50}$6	38546	16123703	418	77	81.89486	86.16667	1.040357	0.468556	0.999747
V_LC5$_{0}$7	44063	18198023	413	79	91.28473	92.90909	1.131113	0.403297	0.999585
